# Inhibition of Gluconeogenesis by Boldine in the Perfused Liver: Therapeutical Implication for Glycemic Control

**DOI:** 10.1155/2023/1283716

**Published:** 2023-04-04

**Authors:** Laís Cristina Lima Silva, Gustavo Henrique de Souza, Vanesa de Oliveira Pateis, Ana Paula Ames-Sibin, Beatriz Paes Silva, Lívia Bracht, Jurandir Fernando Comar, Rosane Marina Peralta, Adelar Bracht, Anacharis Babeto Sá-Nakanishi

**Affiliations:** Department of Biochemistry, Labor of Hepatic Metabolism, State University of Maringá, Maringá, PR, Brazil

## Abstract

The alkaloid boldine occurs in the Chilean boldo tree (*Peumus boldus*). It acts as a free radical scavenger and controls glycemia in diabetic rats. Various mechanisms have been proposed for this effect, including inhibited glucose absorption, stimulated insulin secretion, and increased expression of genes involved in glycemic control. Direct effects on glucose synthesis and degradation were not yet measured. To fill this gap, the present study is aimed at ensuring several metabolic pathways linked to glucose metabolism (e.g., gluconeogenesis) in the isolated perfused rat liver. In order to address mechanistic issues, energy transduction in isolated mitochondria and activities of gluconeogenic key enzymes in tissue preparations were also measured. Boldine diminished mitochondrial ROS generation, with no effect on energy transduction in isolated mitochondria. It inhibited, however, at least three enzymes of the gluconeogenic pathway, namely, phosphoenolpyruvate carboxykinase, fructose-bisphosphatase-1, and glucose 6-phosphatase, starting at concentrations below 50 *μ*M. Consistently, in the perfused liver, boldine decreased lactate-, alanine-, and fructose-driven gluconeogenesis with IC_50_ values of 71.9, 85.2, and 83.6 *μ*M, respectively. Conversely, the compound also increased glycolysis from glycogen-derived glucosyl units. The hepatic ATP content was not affected by boldine. It is proposed that the direct inhibition of hepatic gluconeogenesis by boldine, combined with the increase of glycolysis, could be an important event behind the diminished hyperglycemia observed in boldine-treated diabetic rats.

## 1. Introduction

**Figure 1:**
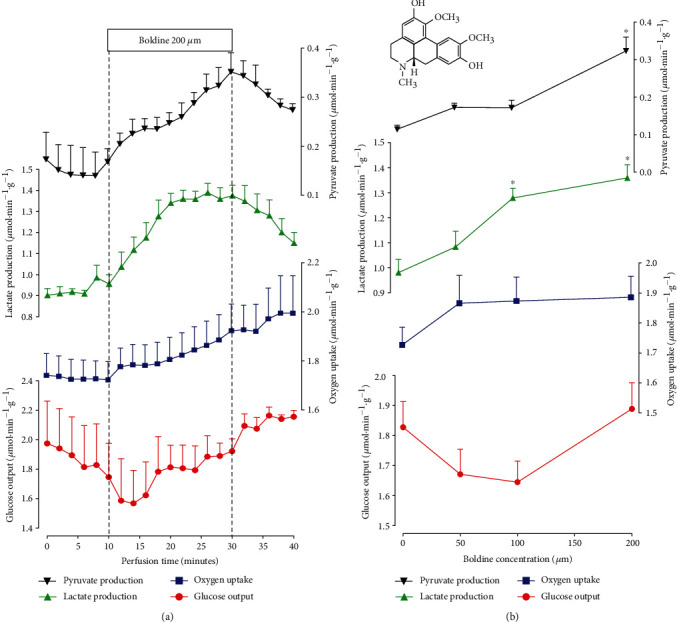
The effects of boldine on glycogen catabolism in the livers from fed rats. (a) Time courses of the changes caused by 200 *μ*M boldine. Data are the means ± mean standard errors of 4 to 6 liver perfusion experiments. The time period of boldine infusion is indicated by the horizontal bar. (b) Concentration dependences of the effects of boldine on the rates of oxygen uptake, lactate and pyruvate productions, and glucose output. All parameters were evaluated before (10 minutes of perfusion time) and at 20 min after starting boldine infusion (30 minutes of perfusion time). The values corresponding to zero boldine concentration (controls) are the means ± mean standard errors of 12 perfusion experiments, computed just before initiating boldine infusion; rates in the presence of boldine represent the means of 4 liver perfusion experiments computed at 30 minutes of perfusion time. Asterisks (^∗^) identify those rates that differ from the control condition, as indicated by the post hoc Student-Newman-Keuls testing (*p* ≤ 0.05).

Boldine is an alkaloid (aporphine class) mainly found in the leaves and bark of the Chilean boldo tree (*Peumus boldus*) (see chemical structure as an inset in the top of Figure 1) widely characterized as an antioxidant [1–5]. Other pharmacological actions, that have also been associated with the ability of boldine of scavenging highly reactive free radicals, include anti-inflammatory, hypoglycemic, antiatherogenic, and antitumoral properties [3, 6–10].

Preparations containing boldine are freely commercialized in the form of gelatin capsules and are popularly used for the treatment of mild dyspepsia (Hebron Pharmaceutic). Boldine-based and boldo-containing pharmaceutical preparations have been used for decades in the treatment of a variety of liver ailments [11]. The hepatoprotective effects of boldine have been demonstrated in animals [3, 12–14] and also in human microsomal membranes [15]. The hepatoprotective actions seem to be related to the antioxidant capacity of boldine, more specifically to its hydroxyl radical scavenger capacity [11] as well as to its ability to inhibit superoxide anion generation by NADPH oxidase in boldine-treated rats [16].

Other studies demonstrated that boldine presents antihyperglycemic action in animals. Jang et al. [10] reported that oral treatment with boldine (100 mg/kg, for 8 weeks) decreased hyperglycemia, lipid peroxidation, and mitochondrial protein carbonylation in the liver, kidney, and pancreas of diabetic rats. Oral treatment with a smaller dose of boldine (50 mg/kg) for 10 weeks was also effective in reducing hyperglycemia and preventing the development of diabetic nephropathy [9]. The dose-dependent reduction of plasma glucose observed in both normal and diabetic rats was correlated with the stimulation of insulin release [17]. Another investigation found that boldine has the ability to lower glucose uptake in isolated intestinal brush-border membrane vesicles or basolateral membrane vesicles as well as glucose absorption during in situ intestinal perfusion [18]. Boldine is also able to exert medium-term effects. For example, in isolated 3T3 cells (differentiated adipocytes), the compound increased adiponectin expression and activated either directly or indirectly the PPAR promoter [19]. Furthermore, boldine oral and intraperitoneal treatment restored endothelial function and prevented both renal and cellular alterations, whose conditions are often impaired in diabetic animals [9, 16]. To our knowledge, however, there are no studies investigating the direct effects of boldine on liver metabolism. This is an important question if one takes into account the general metabolic importance of the liver and its crucial role in the control of glycemia. Additionally, the liver is the place where boldine accumulates upon ingestion and where it also undergoes transformation reactions [2, 20]. For these reasons, this study is aimed at evaluating the direct effect of boldine on carbohydrate metabolism, with a special emphasis on de novo glucose synthesis. Attempts were also made to decipher the mechanisms behind the observed effects.

## 2. Materials and Methods

### 2.1. Chemicals

Boldine (1,10-dimethoxy-2,9-dihydroxy aporphine; 327.37 g/mol), enzymes, and coenzymes were purchased from Sigma-Aldrich Co. (St. Louis, USA). All other chemicals were from the best available grade (98–99.8% purity).

### 2.2. Animals

Male Wistar rats (200–230 g) were used in all experiments. Animals were fed *ad libitum* with a standard laboratory diet (Nuvilab®, Colombo, Brazil). Depending on the protocol, rats were fed or starved for 18 h before the experiments. For all the experiments, the rats were anesthetized by intraperitoneal injection of ketamine (70 mg/kg)+xylazine (7 mg/kg) before removal of the liver. All experiments were done in accordance with the worldwide accepted ethical guidelines for animal experimentation and were previously approved by the Ethics Committee of Animal Experimentation of the State University of Maringá (protocol number 6416011220).

### 2.3. Liver Perfusion

Hemoglobin-free, nonrecirculating perfusion was performed [21, 22]. After cannulation of the portal and cava veins, the liver was positioned in a plexiglass chamber, and the constant flow (provided by a peristaltic pump) was maintained between 30 and 32 mL/min, depending on the liver weight. The perfusion fluid was the Krebs-Henseleit bicarbonate buffer (pH 7.4) containing 25 mg/100 mL bovine serum albumin, saturated with a mixture of oxygen and carbon dioxide (95 : 5) by means of a membrane oxygenator with simultaneous temperature adjustment (37°C). Substrates and boldine were added to the perfusion fluid according to the experimental protocols that are illustrated in each figure. The concentration range was based on previous publications [2, 12]. Due to its low water solubility, boldine was added to the perfusion fluid as a dimethylsulfoxide solution to achieve the desired final concentration (50 to 200 *μ*M). It is already amply documented that dimethylsulfoxide does not significantly affect liver metabolism, at least not when infused at rates up to 32 *μ*L/min [23], a limit that was never surpassed in the present work.

### 2.4. Metabolite Assay

Samples of the effluent perfusion fluid were collected, and the following compounds were assayed by means of standard enzymatic procedures: glucose, lactate, pyruvate, ammonia, and urea [24]. The oxygen concentration in the outflowing perfusate was monitored continuously by polarography [22]. Metabolic rates were calculated from input-output differences, and the total flow rates were referred to the wet weight of the liver.

The hepatic contents of the adenine nucleotides (ATP, ADP, and AMP) were assayed by means of high-performance liquid chromatography (HPLC) in the liver of fasted rats in the presence of lactate as gluconeogenic substrate (control) and lactate plus boldine (200 *μ*M). The liver was clamped in liquid nitrogen at 56 minutes of perfusion time. The liver was weighted and deproteinized, and AMP, ADP, and ATP were detected spectrophotometrically at 254 nm [25]. Identification of the peaks of the investigated compounds was accomplished by comparison of their retention times with those obtained by injecting standards under the same conditions. The concentrations of the compounds were calculated through the regression parameters obtained from the calibration curves. Linear relationships were obtained between the concentrations and the areas under the elution curves.

### 2.5. Tissue Collection and Processing

For isolation of mitochondria and microsomes, fed rats were decapitated, and the livers were removed immediately and cut into small pieces. After homogenization in an isolation medium that consisted of 0.2 M mannitol, 75 mM sucrose, 2 mM Tris-HCl (pH 7.4), 0.2 mM EGTA, and 50 mg/100 mL bovine serum albumin, the mitochondria and microsomes were isolated by differential centrifugation [25–27] and kept at 0–4°C until use. The cytosolic fraction is a supernatant obtained from the centrifugation at 105,000g during the isolation procedure of the microsomal fraction [28].

### 2.6. Mitochondrial ROS Production and Respiration

The rates of mitochondrial ROS production were estimated by measuring the linear increase of fluorescence (504 nm for excitation and 529 nm for emission) due to DCF formation from DCFH-DA via oxidation by H_2_O_2_ in the presence of horseradish peroxidase [29]. The results were expressed as nmol min^−1^ (mg protein)^−1^ and, alternatively, as the effective concentration of boldine that inhibits 50% (IC_50_) of the maximal ROS generation.

Oxygen uptake by freshly isolated mitochondria was measured polarographically using a Teflon-shielded platinum electrode [30]. Pyruvate plus malate (10 mM + 1 mM) and succinate (10 mM) were used as electron donors for complexes I and II, respectively. ADP, for a final concentration of 125 *μ*M, was added at appropriate times. Boldine was added as DMSO solutions with different concentrations to ensure a constant amount of solvent. Controls were run to exclude solvent effects. The final boldine concentrations were in the range between 1 and 200 *μ*M. Rates of oxygen uptake were computed from the slopes of the recorder tracings and expressed as nmol min^−1^ (mg protein)^−1^. The respiration rates were determined in the presence of exogenous ADP (state III) and after ADP exhaustion (state IV) [31]. The protein content was measured using the Folin phenol reagent and bovine serum albumin as standard [32].

### 2.7. Assays of Liver Metabolism-Linked Enzymes

The activity of glucose 6-phosphatase (G6Pase) was measured in isolated microsomes by the spectrophotometric quantification of phosphate released from glucose 6-phosphate [26]. The enzyme activity was calculated based on a standard curve and expressed as nmol of the released phosphate per minute per milligram of protein.

The activities of fructose 1,6-bisphosphatase (FBPase-1) and phosphoenolpyruvate carboxykinase (PEPCK) were determined in the cytosolic fraction. The FBPase-1 activity was measured by spectrophotometric quantification of released phosphate from fructose 1,6-bisphosphate [28]. The PEPCK activity was estimated by coupling the malate dehydrogenase reaction to the PEPCK reaction [33, 34]. The oxidation of NADH by oxaloacetate formed in the PEPCK reaction was followed spectrophotometrically at 340 nm for 5 min, and the enzyme activity was expressed as nmol NADH oxidized per minute per milligram protein.

The pyruvate carboxylase (PC) activity of intact mitochondria was determined by quantifying the incorporation of ^14^C from [^14^C]NaHCO_3_ into components of the tricarboxylic acid cycle. After 10 minutes of incubation at 37°C, the reaction was stopped by adding 0.5 volume of 2 N perchloric acid. Sequentially, the excess [^14^C]NaHCO_3_ was removed by bubbling CO_2_ for 5 minutes, and aliquots were collected to quantify the incorporated radioactivity into acid-stable compounds [33]. The enzyme activity was expressed as nmol of ^14^C per minute per milligram protein.

### 2.8. Data Handling

Statistical analysis was evaluated by variance analysis (ANOVA) with post hoc testing according to Student-Newman-Keuls (*p* ≤ 0.05) using the GraphPad Prism software (version 5.0). Half-maximal effect concentrations (IC_50_) were computed by means of numerical interpolation with Stineman's formula using the Scientist software from MicroMath Scientific Software (Salt Lake City, UT, USA).

## 3. Results

### 3.1. Glycogen Catabolism, Glycolysis, and Oxygen Uptake

The first experiments were planned to evaluate the effects of boldine on glycogen catabolism and glycolysis. For this purpose, the livers of fed rats were used, as this ensures high levels of hepatic glycogen [34, 35]. The results are shown in Figure 1.  Figure 1(a) illustrates the time course of the action of 200 *μ*M boldine on glucose output, lactate, and pyruvate production in addition to oxygen uptake. It also illustrates the experimental protocol that was followed in these specific experiments. Boldine infusion was started at 10 minutes of perfusion time and continued for the next 20 minutes, i.e., until 30 minutes of perfusion time. At this time, the infusion of boldine was interrupted, but samples were still collected during the following 10 minutes. Lactate and pyruvate productions were increased when boldine was introduced. Cessation of boldine infusion promoted a return to the basal levels, although this return was still incomplete at the time when the perfusion was interrupted. Oxygen output presented an increasing tendency during boldine infusion, and glucose showed an initial transient decreasing tendency. The same experimental protocol was used when boldine was infused at concentrations of 50 and 100 𝜇M. The time courses of these experiments are not shown, but Figure 1(b) presents the concentration dependences of the effects of boldine. The values presented in this graph are the rates before starting boldine infusion (corresponding to zero boldine concentration) and the rates reached after stabilization of the changes induced by each boldine concentration (30 minutes of perfusion time). Lactate production started to increase at the concentration of 100 𝜇M. Pyruvate production, in turn, was significantly increased only at the concentration of 200 𝜇M. The simultaneous increases in lactate production and pyruvate production resulted in no modifications in the lactate/pyruvate ratio. Finally, no statistically significant modifications in the rates of glucose output and oxygen uptake were found in spite of the presence of increasing (oxygen uptake) and decreasing tendencies (glucose output).

### 3.2. Lactate Gluconeogenesis

**Figure 2:**
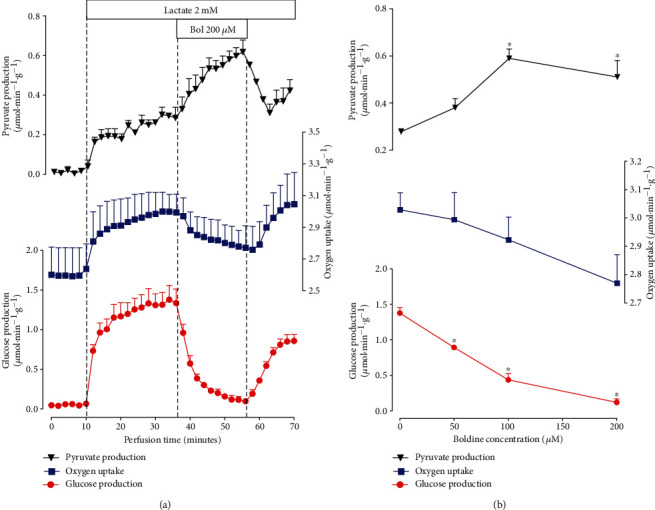
Effect of boldine on the lactate gluconeogenesis in the perfused liver isolated from fasted rats. Time courses (a) and concentration-dependent effects of boldine (b) on lactate gluconeogenesis and related parameters in the livers isolated from fasted rats. Liver was perfused as described in the Materials and Methods. In (a), data are the means ± mean standard errors of 3 to 4 liver perfusion experiments. Boxes near the time scale indicate the lactate and boldine infusion periods and concentrations. In (b), the rates of pyruvate and glucose productions and oxygen uptake were evaluated at 36 minutes of perfusion time (control condition, zero boldine concentration; *n* = 10) and at 56 min perfusion time (20 minutes after starting boldine infusion; *n* = 4). Asterisks (^∗^) in (b) indicate those rates that differ from the control condition (absence of boldine; *p* ≤ 0.05).

Livers from 18 h fasted rats were perfused to ensure low glycogen levels. Under this condition, glucose output reflects mainly the rate of gluconeogenesis [35].  Figure 2(a) shows the time course of the changes caused by boldine (200 *μ*M) in the presence of lactate (2 mM) as substrate. After preperfusion with the substrate-free Krebs-Henseleit buffer (basal levels), lactate infusion produced rapid increases in glucose and pyruvate productions and in oxygen uptake, with subsequent stabilization at about 34 minutes of perfusion time. The infusion of boldine at 36 minutes of perfusion time resulted in a progressive decrease in glucose production, achieving complete inhibition (basal levels) at 56 minutes of perfusion time. This inhibition was partially reversed upon cessation of boldine infusion. Oxygen uptake experienced a slight diminution, but the significance is doubtful because the variations do not exceed the corresponding standard errors. Pyruvate production, on the other hand, was progressively elevated by boldine, an elevation that was completely abolished upon cessation of the boldine infusion. The same protocol was followed with two other concentrations, and the effects versus concentration relationship are illustrated in Figure 2(b). Glucose production was progressively reduced with increasing boldine concentrations. Half-maximal inhibition of glucose production, as revealed by numerical interpolation, can be expected at a boldine concentration of 71.85 *μ*M. Pyruvate production, on the other hand, was elevated at 100 𝜇M and maintained until 200 𝜇M. For oxygen uptake, on the other hand, there was a tendency toward diminution when the boldine concentration was increased. However, no statistical significance was found.

### 3.3. Fructose Metabolism

**Figure 3:**
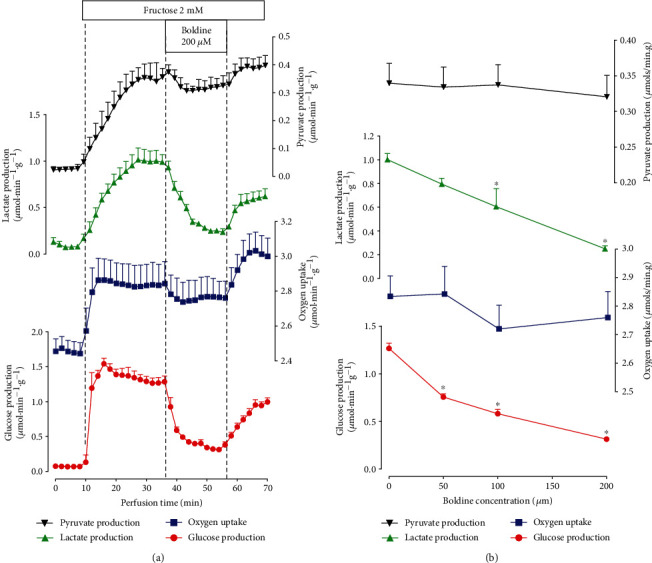
Effects of boldine on fructose gluconeogenesis in the perfused livers isolated from fasted rats. Time courses (a) and concentration-dependent effects of boldine (b) on fructose gluconeogenesis and related parameters. Livers were perfused as described in the Materials and Methods. In (a), data are the means ± mean standard errors of 4 liver perfusion experiments. Boxes near to the time scale indicate the fructose and boldine infusion periods and concentrations. In (b), the rates of pyruvate, lactate, and glucose productions and oxygen uptake were evaluated at 36 minutes of perfusion time (control condition, zero boldine concentration; *n* = 12) and at 56 min perfusion time (*n* = 4). Asterisks (^∗^) in (b) indicate those rates that differ from the control condition (absence of boldine; *p* ≤ 0.05).

In the liver, fructose metabolism can undergo both transformation into glucose (anabolic pathway) and catabolic degradation into pyruvate and lactate (fructolysis). Fructose transformation into glucose uses only a fraction of the enzymes needed for lactate gluconeogenesis. The experimental protocol with fructose as substrate was similar to that one already described for the experiments with lactate. Here again, the livers from fasted rats were used in order to avoid interference by endogenous glycogen [34, 35].  Figure 3(a) illustrates the time course of the modifications induced by 200 𝜇M boldine infusion. Fructose infusion resulted, as expected, in pronounced increases in glucose, pyruvate, and lactate productions and oxygen uptake (stimulated state). The introduction of boldine (200 𝜇M) at 36 minutes of perfusion time produced a fast diminution of glucose production that was almost completed at 56 minutes of perfusion time. Oxygen uptake, on the other hand, presented an inhibitory tendency whose significance is doubtful due to its small extent (less than 3% of the stimulated state) and high standard error. Lactate production, an indicator of the catabolic breakdown of fructose, was clearly inhibited by boldine, achieving a new steady state in the final part of the boldine infusion period. Pyruvate production presented a tendency toward inhibition, whose significance was lacking. After cessation of boldine infusion, all parameters tended to return to the levels that were found before the onset of boldine infusion (stimulated state). Particularly, oxygen uptake showed a transient stimulation after stopping boldine infusion.

The same protocol was repeated with two other boldine concentrations, and the new steady states induced by each boldine concentration are represented in Figure 3(b). Inhibition of glucose production shows a well-defined concentration dependence, and 50% inhibition can be expected at the concentration of 83.62 *μ*M. Oxygen uptake was not significantly modified by the various boldine concentrations the same being valid for pyruvate production. In the case of lactate production, however, a concentration-dependent inhibition was found with an IC_50_ of 128.5 *μ*M.

### 3.4. Glycerol Gluconeogenesis

**Figure 4:**
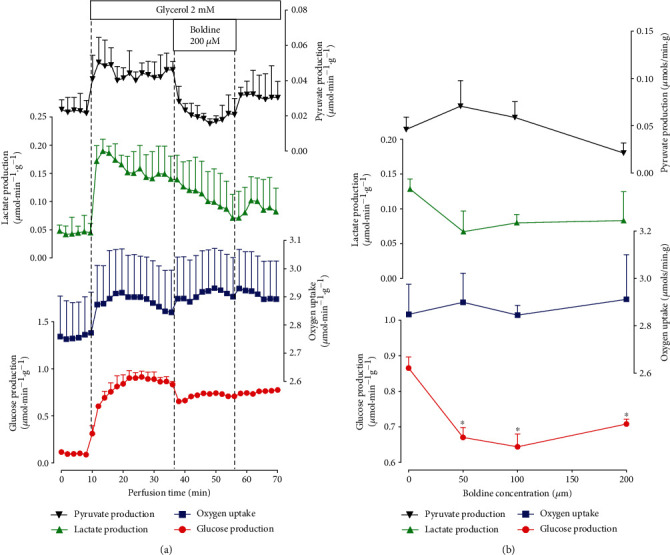
Effect of boldine on glycerol gluconeogenesis in the perfused livers isolated from fasted rats. (a) The time courses of the effects of glycerol and 200 *μ*M boldine and (b) the concentration dependences of the effects of boldine. Livers were perfused as described in the Materials and Methods. In (a), data are the means ± mean standard errors of 3 to 4 liver perfusion experiments. Boxes near to the time scale indicate the glycerol and boldine infusion periods and concentrations. In (b), the rates of pyruvate, lactate, and glucose productions and oxygen uptake were evaluated at 36 minutes of perfusion time (control condition; *n* = 12) and at 56 min perfusion time (*n* = 4). Asterisks (^∗^) in (b) indicate statistically significant differences (*p* ≤ 0.05) when compared to the control condition (absence of boldine), as given by the post hoc Student-Newman-Keuls testing.

In order to focus further the investigations on limited portions of the gluconeogenic pathway, the action of boldine on glycerol-driven glucose production was measured. The experimental protocol was the same as that one used with other gluconeogenic substrates and is illustrated in Figure 4(a). The livers were from fasted rats. The results are illustrated in Figure 4(a). As expected, glycerol infusion stimulated glucose, pyruvate, and lactate productions and oxygen uptake. Boldine tended to diminish glucose and pyruvate production.  Figure 4(b) presents the concentration dependences of the boldine effects. Glucose production was significantly inhibited to similar extents by all three concentrations. This inhibition amounted to approximately 20%. Oxygen uptake and lactate and pyruvate productions, however, suffered no significant modifications.

### 3.5. Alanine Metabolism

**Figure 5:**
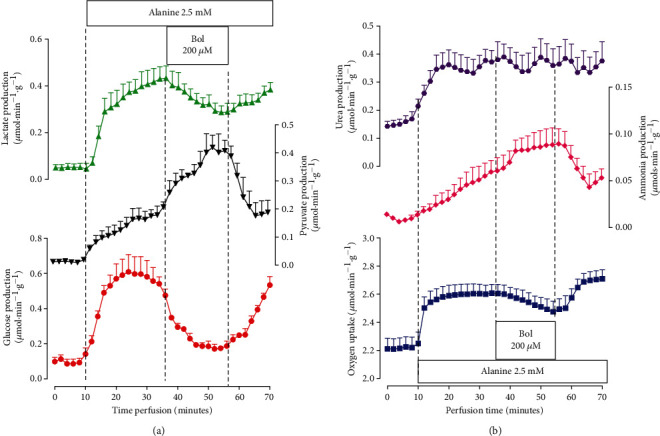
Time course of the effects of boldine on the alanine gluconeogenesis in the perfused livers isolated from fasted rats. Livers were perfused as described in the Materials and Methods. Boxes near to the time scale indicate the alanine and boldine infusion periods and concentrations. The outflowing perfusate was sampled every 2 minutes and used to quantify glucose, pyruvate, lactate, ammonia, and urea. Oxygen uptake was monitored polarographically by a platinum electrode. Data are the means ± mean standard errors of 4 to 5 liver perfusion experiments.

**Figure 6:**
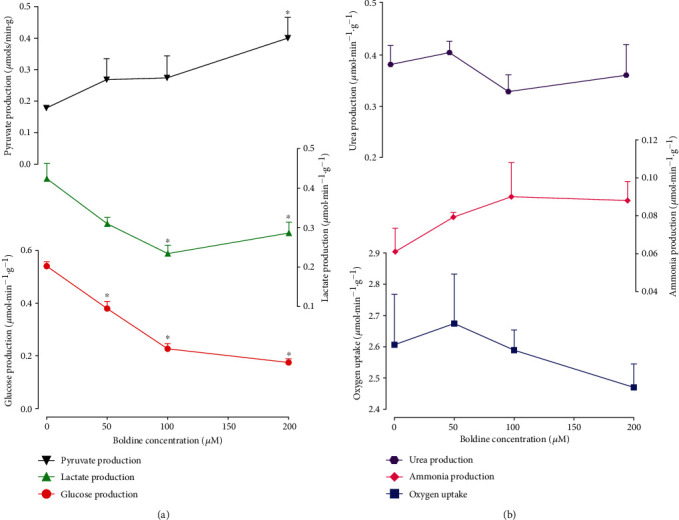
Concentration dependence of the effects of boldine on alanine gluconeogenesis in the perfused livers from fasted rats. Livers were perfused as described in the Materials and Methods. The metabolic rates were calculated at 36 minutes of perfusion time (control condition, zero boldine concentration; *n* = 12) and at 5 minutes of perfusion time (*n* = 4). Asterisks (^∗^) indicate statistically significant differences when compared to the control condition (absence of boldine), as indicated by post hoc testing according to Student-Newman-Keuls (*p* ≤ 0.05).

In order to investigate the possible effects of boldine on nitrogen metabolism, alanine was infused. This procedure allows determining simultaneously ureagenesis and ammoniagenesis in addition to gluconeogenesis. The experiments were conducted with the livers from fasted rats, and the results are shown in Figure 5. Alanine induces a more oxidized state when compared to lactate, and the transfer of the amine group also influences the urea cycle and several related pathways. Alanine infusion quickly increased glucose, pyruvate, lactate, ammonia, and urea productions and oxygen consumption. These increases reached steady states at 26 min perfusion time. Boldine inhibited glucose production almost completely with a new steady state at 46 min perfusion time. Lactate production was clearly diminished, and pyruvate increased. The other parameters, namely, oxygen uptake, ammonia production, and urea production, were only slightly affected, the variations not exceeding the corresponding standard errors. The concentration dependences of the boldine effects on alanine metabolism are shown in Figure 6. Inhibition of glucose production presented a clear concentration dependence, with 50% inhibition at the concentration of 85.2 *μ*M. Boldine elevated pyruvate production, an effect that was significant only at the 200 𝜇M concentration. Lactate production was inhibited by almost 50% at the 200 𝜇M concentration. Nitrogen catabolism (ammonia and urea productions) was practically not affected by boldine, the same being valid for oxygen uptake.

### 3.6. Effects of Boldine on the Activities of Gluconeogenesis Rate-Limiting Enzymes, Mitochondrial Respiration, ROS Generation, and Levels of Adenosine Nucleotides

**Figure 7:**
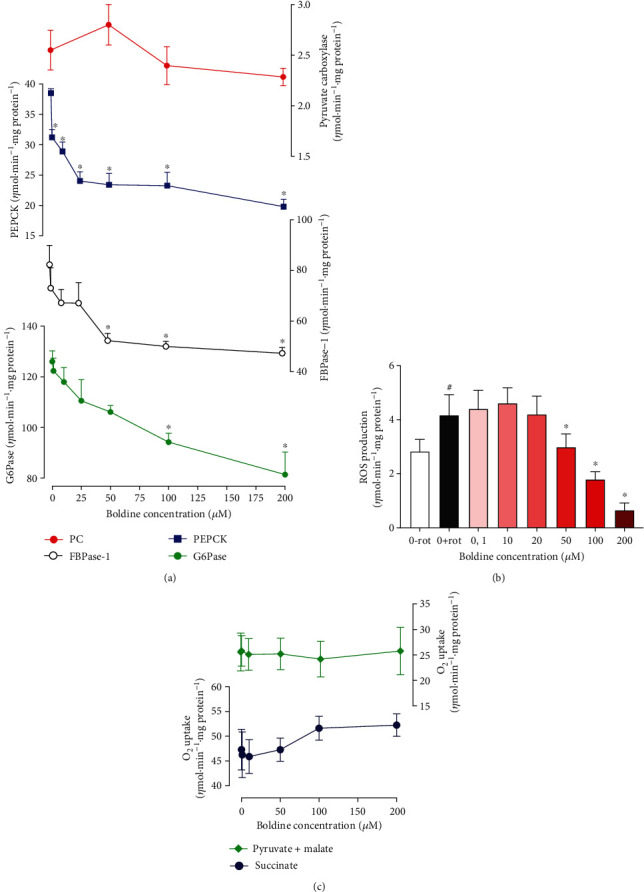
Concentration dependences of the effects of boldine on regulatory enzymes of the gluconeogenic pathway (a), ROS production (b), and mitochondrial respiration (c). Experimental procedures were described in the Materials and methods. (a) Values are means ± mean standard errors of 4 to 6 assays. Asterisks (^∗^) indicate statistical significance in comparison with the control condition (Student-Newman-Keuls, *p* ≤ 0.05). (b, c) Mitochondria of the rat livers were isolated as described in the Materials and methods. Values are means ± mean standard errors of 4 to 6 assays. In (b), 0-rot: without boldine and rotenone; 0+rot: without boldine in the presence of rotenone; all boldine concentrations were evaluated in the presence of rotenone. The symbol # indicates statistical significance in comparison with 0-rot; ^∗^ indicates statistical significance in comparison with the stimulated condition (0+rot) (Student-Newman-Keuls, *p* ≤ 0.05).

In order to investigate a possible direct effect of boldine on rate-limiting enzymes of gluconeogenesis, several assays were performed in cellular fractions of the liver.  Figure 7(a) shows the activities that were measured as functions of the boldine concentrations. Boldine inhibited the activities of glucose 6-phosphatase, fructose 1,6-bisphosphatase, and phosphoenolpyruvate carboxykinase in a concentration-dependent manner, with 35, 42, and 49% inhibition, respectively, at the concentration of 200 *μ*M. The fructose 1,6-bisphosphatase concentration dependence suggests that inhibition is of the incomplete type, as no substantial increment was found when the boldine concentration was increased from 100 to 200 *μ*M. Pyruvate carboxylase, finally, was not inhibited by boldine.

Figure 7(b) shows that the mitochondrial ROS generation was inhibited by boldine. This inhibition started at the concentration of 50 *μ*M and achieved 84% at 200 *μ*M. Interpolation predicts that 50% inhibition can be expected to occur at the concentration of 84.8 *μ*M.  Figure 7(c), on the other hand, shows that boldine did not interfere with the mitochondrial respiration nor did it affect the ADP/O ratios (not shown), at least not when succinate or pyruvate+malate were the substrates.

The adenine nucleotide content of the liver in the presence of boldine is listed in Table 1. The data reveal that the compound had no influence at all on the levels of these compounds, excluding thus any direct effect of the compound on hepatic energy metabolism.

## 4. Discussion

**Figure 8:**
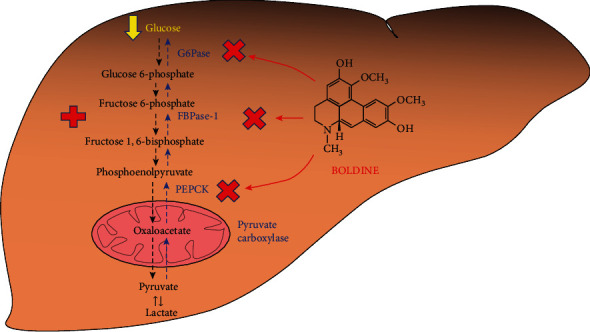
Schematic representation of the main sites of action of boldine on the pathways leading to glucose synthesis and degradation in the liver. The + sign denotes stimulation, and the x denotes inhibition.

The results of the present work reveal that boldine is able to modify several metabolic pathways linked to carbohydrate metabolism in the perfused liver. The major findings were (1) increased glycolysis from liver glycogen, (2) substantial inhibition of gluconeogenesis from several substrates, and (3) inhibition of fructolysis. All these effects were not associated with significant modifications in the hepatic ATP content nor with impaired mitochondrial respiration. Additionally, boldine diminished ROS production in isolated liver mitochondria. Modifications in metabolic fluxes result from multiple actions. Most of them are summarized in Figure 8 in order to facilitate understanding. Altogether, these effects confirm the beneficial role of boldine as a modulator of glucose homeostasis and as a potent antioxidant in the liver, phenomena that can significantly contribute to the diminished blood hyperglycemia that was described in diabetic animals treated with this alkaloid [9, 10, 17].

Stimulation of the glycolytic pathway was demonstrated when the livers from fed rats were perfused. Under this condition, boldine expressively stimulated lactate and pyruvate productions from endogenous glycogen. This is an important finding because the enhancement of glycolysis is generally regarded as an efficient way of lowering plasma glucose and consequently controlling hyperglycemia [36]. Stimulation of glycolysis occurs often as a compensatory phenomenon for impaired ATP production in mitochondria. However, our results discard this possibility, and therefore, other targets must be involved such as the activities of phosphofructokinase and pyruvate kinase, key enzymes of the glycolytic pathway. Another reasonable explanation would be a possible increase in fructose 2,6-biphosphate levels during the drug infusion period. An increase in fructose 2,6-biphosphate would stimulate phosphofructokinase, which in turn would lead to increased glycolysis, and inhibit the fructose 1,6-bisphosphatase activity, contributing to the inhibition of gluconeogenesis that was also observed in the present work. This hypothesis gains importance when one considers the demonstration that the glycemic control drugs troglitazone, glipizide, and tolbutamide increase the concentration of fructose 2,6-biphosphate in isolated rat hepatocytes and that this increase inversely correlates with the rate of gluconeogenesis [37–39].

The hypothesis of an increase in fructose 2,6-phosphate, however, would not completely explain the observed inhibition of gluconeogenesis, since it has been demonstrated that alterations in this metabolite do not have dramatic effects on gluconeogenesis from lactate [40]. Therefore, studies were subsequently conducted to determine which specific regions of the gluconeogenic pathways were responsible for this inhibition. The data that were obtained in the experiments using fructose, alanine, and glycerol as gluconeogenic substrates suggest multiple sites of inhibition. The magnitudes of gluconeogenesis inhibition can be inferred from the half-maximal inhibitory concentration (IC_50_). Half-maximal inhibition of lactate and alanine gluconeogenesis occurred at boldine concentrations of 71.8 *μ*M and 85.2 *μ*M, respectively. These are similar concentrations. The first step of lactate and alanine metabolization in the liver is their conversion into pyruvate, catalyzed by two different enzymes, lactate dehydrogenase, and alanine aminotransferase, respectively. Inhibition of the net flux through these two enzymes at almost exactly the same degree by boldine is not a very likely event. Furthermore, these enzymes operate under near-equilibrium conditions in the liver, requiring, thus, very high inhibition degrees for exerting a significant influence on the gluconeogenic pathway. It is important to note that when the livers were supplied with lactate or alanine, pyruvate overflow was slightly increased at the highest boldine concentration. The phenomenon suggests that a step situated downstream to pyruvate generation is more likely to be inhibited by boldine. One such step could be the reaction catalyzed by the phosphoenolpyruvate carboxykinase (PEPCK), which our experiments revealed indeed to be inhibited by boldine. PEPCK catalyzes the conversion of oxaloacetate into phosphoenolpyruvate. Although the metabolic control of this enzyme over gluconeogenesis is low under normal conditions [41], inhibition can change the control strength of any enzyme. In fact, as shown for the liver of db/db mice, acute inhibition of PEPCK was sufficient to improve glycemia [42].

Even so, it is unlikely that the inhibition of PEPCK is the sole responsible for the inhibition of gluconeogenesis. Fructose transformation into glucose was also substantially inhibited with an IC_50_ of 83.62 *μ*M, which is comparable to that one found for alanine or lactate, and the pathway that leads to its transformation into glucose is situated downstream to the step catalyzed by PEPCK. Furthermore, glycerol gluconeogenesis, which uses in part the same pathway as fructose gluconeogenesis was also inhibited, though to a much lesser extent (maximally 20%). This combination of observations suggests not only that there must be other steps that can be inhibited by boldine in addition to the PEPCK step but also that there must be a step whose inhibition specifically affects fructose metabolism. Additional enzymatic steps sensitive to boldine were indeed found in the present work, more specifically FBPase-1 and G6Pase. G6Pase, an enzyme found mainly in the liver, plays the important role of providing glucose during starvation. G6Pase catalyzes the last step of gluconeogenesis, and its inhibition could partly explain the reduction of gluconeogenesis. However, as already demonstrated [43], even a strong inhibition of glucose 6-phosphatase causes only a transitory change in the rates of glucose output by the liver because the resulting increased concentration of glucose 6-phosphate largely restores the rate of its own hydrolysis. The inhibition of FBPase-1 can also be pointed out as an additional mechanism able to contribute to the reduced gluconeogenic flux, not only for fructose and glycerol gluconeogenesis but also for lactate and alanine gluconeogenesis as well. What the inhibition of FBPase-1 and G6Pase cannot explain, however, is the great difference between the diminutions caused by boldine in the rates of glucose production from fructose and glycerol. Hypotheses are clearly needed in this particular respect. A hypothesis that has some observational support is that boldine could be interfering with another particular step of the pathway of fructose metabolism, for example, that one catalyzed by fructokinase. This is a hypothesis supported by the observed inhibition of fructolysis (lactate+pyruvate productions), but additional experimental work is necessary to clarify this question.

In addition, we cannot discard a possible diversion of glucose 6-phosphate for boldine transformation. Boldine's hepatic biotransformation occurs mainly by O-conjugation with glucuronate and sulphate and, to a lesser extent, via N-demethylation [20]. The latter is catalyzed by the microsomal system (CYP450) which requires NADPH as the source of electrons. Glucuronidation, in turn, requires the availability of glucose to synthesize glucuronic acid. In the liver, the main source of glucuronic acid is normally glycogenolysis, but in the absence of glycogen, gluconeogenesis is the sole possible source [34, 44]. In this sense, the diversion of a fraction of glucose 6-phosphate or malate into the synthesis of NADPH and glucuronic acid is a likely phenomenon that could partially contribute for reducing hepatic gluconeogenesis.

Direct inhibition of ROS generation in mitochondria by boldine is certainly a beneficial effect, corroborating previous publications [10]. Several *in vitro* studies have revealed that boldine is a very efficient scavenger of the hydroxyl radical (HO•) and peroxyl radical [3, 10, 45]. Contrasting with the observations of Jang et al. [10], which found inhibition of ROS production at boldine concentrations as low as 1 *μ*M, our results showed significant inhibition starting at 50 *μ*M. These differences can possibly be attributed to the distinct assays used for measuring mitochondrial ROS generation. Jang et al. [10] quantified the ROS content after 30 minutes of reaction. In our experiments, the production of ROS was followed continuously by means of a kinetic assay, in which real-time ROS production was measured. The direct antioxidant effect of boldine in mitochondria occurs without simultaneous changes in oxygen uptake in isolated mitochondria as well as in the perfused liver. Our results agree with those of Jiménez and Speisky [2], who showed that oxygen consumption remained relatively constant during 90 minutes of boldine perfusion. Furthermore, these authors also reported that during the whole perfusion time, no leakage of LDH activity occurs into the outflowing perfusion fluid, showing that boldine does not damage the hepatic tissue. In accordance with this, it has also been previously shown that boldine prevents mitochondrial dysfunction induced by thioacetamide [12].

It is worth mentioning that metformin, a classical drug for glycemic control, also reduced hepatic gluconeogenesis in the perfused liver [46]. Differently to boldine, the inhibition of gluconeogenesis by metformin results mainly from an inhibition of the mitochondrial respiration as also from an indirect FBPase-1 inhibition [47]. However, the metformin active concentration in these experiments was above 1 mM. This concentration exceeds the active concentrations of boldine in the present study by a factor of 20, as this compound began to block lactate gluconeogenesis at the concentration of 50 *μ*M. Therefore, the fact that boldine inhibits gluconeogenesis at relatively low concentrations, besides not modifying mitochondrial energy transduction, makes it a potential molecule for being used in the therapy of diabetes.

Absorption of boldine into plasma is rapid, and an average *C*_max_ of 31 *μ*M is reached after the administration of 20 mg/kg (intravenously) in rats [48]. However, this alkaloid concentrates in hepatocytes where it can reach higher levels in relation to blood. For instance, the maximal concentration of boldine in the rat liver was 72 and 88 𝜇M after oral administration of 50 and 75 mg/kg, respectively [2]. In addition to that, it was demonstrated that boldine metabolization by the perfused liver is not constant but tends to decrease at higher portal concentrations [2], which suggests that repeated doses or high doses of boldine can increase its portal and plasma concentrations. So, it is possible that concentrations in the range of 50 to 200 *μ*M (that were those used in the present study) are easily achieved, or even surpassed in hepatocytes. Available data in isolated cells and various animal models point out that boldine presents low toxicity [3], but its actual innocuousness in humans still remains to be established. There are no data concerning the pharmacological doses of boldine in humans. However, according to the translation formula of Reagan-Shaw et al. [49], effective doses in rats of 50 and 75 mg/kg can be translated in humans into 8 and 12 mg/kg, respectively.

In conclusion, the inhibition of gluconeogenesis by boldine in the perfused liver is probably the consequence of the inhibition of several rate-limiting enzymes with a possible minor contribution of the deviation of glucosyl units for the glucuronidation reactions. The capacity of boldine to modulate glucose metabolism in the liver by inhibiting gluconeogenesis and stimulating glycolysis and inhibiting mitochondrial ROS generation, associated with the lack of interference with mitochondrial respiration, makes this alkaloid a potential therapeutic tool for glycemic control.

## Figures and Tables

**Table 1:** Effects of boldine (200 *μ*M) on the adenine mononucleotide contents of the livers from fasted rats. The liver perfusion and the analytical procedures were described in the Materials and methods. In control livers, 2 mM L-lactate was infused alone from 10 to 56 minutes; the test livers were perfused with 2 mM lactate from 10 to 36 minutes and with 200 *μ*M boldine from 36 to 56 minutes. At 56 minutes, the livers were freeze-clamped in liquid nitrogen, and the adenine nucleotides were extracted with cold perchloric acid. Values are means ± mean standard errors of 4 to 5 experiments. Asterisks (^∗^) indicate statistical significance in comparison with the control condition according to the Student-Newman-Keuls post hoc testing (*p* ≤ 0.05).

	Control	Boldine
ATP	0.90 ± 0.045 (*n* = 6)	1.04 ± 0.05 (*n* = 7)
ADP	0.86 ± 0.03 (*n* = 6)	0.89 ± 0.03 (*n* = 7)
AMP	0.53 ± 0.09 (*n* = 6)	0.44 ± 0.04 (*n* = 5)
SOMA (ATP+ADP+AMP)	2.30 ± 0.05 (*n* = 6)	2.34 ± 0.11 (*n* = 5)
ATP/ADP	1.06 ± 0.06 (*n* = 6)	1.20 ± 0.08 (*n* = 8)
ATP/AMP	1.73 ± 0.13 (*n* = 6)	2.29 ± 0.21 (*n* = 5)^∗^

## Data Availability

Data are available on request.

## References

[1] Speisky H., Cassels B. K., Lissi E. A., Videla L. A. (1991). Antioxidant properties of the alkaloid boldine in systems undergoing lipid peroxidation and enzyme inactivation. *Biochemical Pharmacology*.

[2] Jiménez I., Speisky H. (2000). Biological disposition of boldine: in vitro and in vivo studies. *Phytotherapy Research*.

[3] O’Brien P., Carrasco-Pozo C., Speisky H. (2006). Boldine and its antioxidant or health-promoting properties. *Chemico-Biological Interactions*.

[4] Muthna D., Cmielova J., Tomsik P., Rezacova M. (2013). Boldine and related aporphines: from antioxidant to antiproliferative properties. *Natural Product Communications*.

[5] Nabavi S. M., Uriarte E., Fontenla J. A., Rastrelli L., Sobarzo-Sanchez E. (2017). Aporphine alkaloids and their antioxidant medical application: from antineoplastic agents to motor dysfunction diseases. *Current Organic Chemistry*.

[6] Ezhilarasan D., Raghunandhakumar S. (2021). Boldine treatment protects acetaminophen-induced liver inflammation and acute hepatic necrosis in mice. *Journal of Biochemical and Molecular Toxicology*.

[7] Bannach R., Valenzuela A., Cassels B. K., NúnezVergara L. J., Speisky H. (1996). Cytoprotective and antioxidant effects of boldine on tert-butyl hydroperoxide-induced damage to isolated hepatocytes. *Cell Biology and Toxicology*.

[8] Gotteland M., Jimenez I., Brunser O. (1997). Protective effect of boldine in experimental colitis. *Planta Medica*.

[9] Hernández-Salinas R., Vielma A. Z., Arismendi M. N., Boric M. P., Sáez J. C., Velarde V. (2013). Boldine prevents renal alterations in diabetic rats. *Journal Diabetes Research*.

[10] Jang Y. Y., Song J. H., Shin Y. K., Han E. S., Lee C. S. (2000). Protective effect of boldine on oxidative mitochondrial damage in streptozotocin-induced diabetic rats. *Pharmacological Research*.

[11] Speisky H., Cassels B. K. (1994). Boldo and boldine: an emerging case of natural drug development. *Pharmacological Research*.

[12] Heidari R., Arabnezhad M. R., Ommati M. M., Azarpira N., Ghodsimanesh E., Niknahad H. (2019). Boldine supplementation regulates mitochondrial function and oxidative stress in a rat model of hepatotoxicity. *Pharmaceutical Sciences*.

[13] Subramaniam N., Kannan P., Thiruvengadam D. (2019). Hepatoprotective effect of boldine against diethylnitrosamine-induced hepatocarcinogenesis in Wistar rats. *Journal of Biochemical and Molecular Toxicology*.

[14] Heidari R., Moezi L., Asadi B., Ommati M. M., Azarpira N. (2017). Hepatoprotective effect of boldine in a bile duct ligated rat model of cholestasis/cirrhosis. *PharmaNutrition*.

[15] Kringstein P., Cederbaum A. I. (1995). Boldine prevents human liver microsomal lipid peroxidation and inactivation of cytochrome P4502E1. *Free Radical Biology & Medicine*.

[16] Lau Y. S., Tian X. Y., Huang Y., Murugan D., Achike F. I., Mustafa M. R. (2013). Boldine protects endothelial function in hyperglycemia-induced oxidative stress through an antioxidant mechanism. *Biochemical Pharmacology*.

[17] Chi T. C., Lee S. S., Su M. J. (2006). Antihyperglycemic effect of aporphines and their derivatives in normal and diabetic rats. *Planta Medica*.

[18] Nguyen K. H., Ta T. N., Pham T. H. M. (2012). Nuciferine stimulates insulin secretion from beta cells-an in vitro comparison with glibenclamide. *Journal of Ethnopharmacology*.

[19] Yu B., Cook C., Santanam N. (2009). The aporphine alkaloid boldine induces adiponectin expression and regulation in 3T3-L1 cells. *Journal of Medicinal Food*.

[20] Hroch M., Micuda S., Cermanová J., Chládek J., Tomšík P. (2013). Development of an HPLC fluorescence method for determination of boldine in plasma, bile and urine of rats and identification of its major metabolites by LC-MS/MS. *Journal of Chromatography B*.

[21] Scholz R., Bücher T., Chance B., Estabrook R. W., Williamson J. R. (1965). Hemoglobin-free perfusion of rat liver. *Control of Energy Metabolism*.

[22] Kelmer-Bracht A. M., Ishii E. L., Andrade P., Bracht A. (1984). Construction of a liver perfusion apparatus for studies on metabolic regulation and mechanisms of drug action. *Brazilian Archives of Biology and Technology*.

[23] Acco A., Comar J. F., Bracht A. (2004). Metabolic effects of propofol in the isolated perfused rat liver. *Basic & Clinical Pharmacology & Toxicology*.

[24] Bergmeyer H. U. (1974). *Methods of Enzymatic Analysis*.

[25] Maldonado M. R., Bracht L., Sa-Nakanishi A. B. (2018). Actions of p-synephrine on hepatic enzyme activities linked to carbohydrate metabolism and ATP levels in vivo and in the perfused rat liver. *Cell Biochemistry and Function*.

[26] Vilela V. R., Oliveira A. L., Comar J. F., Peralta R. M., Bracht A. (2014). Tadalafil inhibits the cAMP stimulated glucose output in the rat liver. *Chemico-Biological Interactions*.

[27] Bracht A., Ishii-Iwamoto E. L., Salgueiro-Pagadigorria C. L., Bracht A., Ishii-Iwamoto E. L. (2003). O estudo do metabolismo energético em mitocôndrias isoladas de tecido animal. *Métodos de Laboratório Em Bioquímica*.

[28] Sá-Nakanishi A. B., de Oliveira M. C., Pateis V. O. (2020). Glycemic homeostasis and hepatic metabolism are modified in rats with global cerebral ischemia. *Biochimica et Biophysica Acta (BBA)-Molecular Basis of Disease*.

[29] Biazon A. C. B., Wendt M. M. N., Moreira J. R. (2016). The in vitro antioxidant capacities of hydroalcoholic extracts from roots and leaves of Smallanthus sonchifolius (yacon) do not correlate with their in vivo antioxidant action in diabetic rats. *Journal of Biosciences and Medicines*.

[30] Voss D. O., Campello A. P., Bacila M. (1961). The respiratory chain and the oxidative phosphorylation of rat brain mitochondria. *Biochemical and Biophysical Research Communications*.

[31] Sá-Nakanishi A. B., Soni-Neto J., Moreira L. S. (2018). Anti-inflammatory and antioxidant actions of methyl jasmonate are associated with metabolic modifications in the liver of arthritic rats. *Oxidative Medicine and Cellular Longevity*.

[32] Lowry O. H., Rosebrough N. J., Farr A. L., Randall R. J. (1951). Protein measurement with the Folin phenol reagent. *The Journal of Biological Chemistry*.

[33] Bracht L., Caparroz-Assef S. M., Bracht A., Bersani-Amado C. A. (2016). Effect of the combination of ezetimibe and simvastatin on gluconeogenesis and oxygen consumption in the rat liver. *Basic & Clinical Pharmacology & Toxicology*.

[34] Pereira-Maróstica H. V., Bracht L., Comar J. F., Peralta R. M., Bracht A., Sá-Nakanishi A. B. (2022). The rapid transformation of triclosan in the liver reduces its effectiveness as inhibitor of hepatic energy metabolism. *Toxicology and Applied Pharmacology*.

[35] Comar J. F., de Oliveira D. S., Bracht L., Kemmelmeier F. S., Peralta R. M., Bracht A. (2016). The metabolic responses to L-glutamine of livers from rats with diabetes types 1 and 2. *PLoS One*.

[36] Wu C., Okar D. A., Newgard C. B., Lange A. J. (2001). Overexpression of 6-phosphofructo-2-kinase/fructose-2,6-bisphosphatase in mouse liver lowers blood glucose by suppressing hepatic glucose production. *The Journal of Clinical Investigation*.

[37] Cabello M. A., Monge L., Ortega J. L., Samper B., Feliu J. E. (1987). Effect of glipizide on hepatic fructose 2,6-bisphosphate concentration and glucose metabolism. *Metabolism*.

[38] Marano K., Inoue Y., Emot M., Kaku K., Kaneko T. (1994). CS-045, a new oral antidiabetic agent, stimulates fructose-2,6-bisphosphate production in rat hepatocytes. *European Journal of Pharmacology*.

[39] Foster S. E., Raman P., Judd R. L. (1998). Effect of troglitazone (Rezulin) on fructose 2,6-bisphosphate concentration and glucose metabolism in isolated rat hepatocytes. *Diabetes*.

[40] Hue L., Bartron R. (1984). Role of fructose-2,6-bisphosphate in the control by glucagon of gluconeogenesis from various precursors in isolated rat hepatocytes. *The Biochemical Journal*.

[41] Burgess S. C., He T., Yan Z. (2007). Cytosolic phosphoenolpyruvate carboxykinase does not solely control the rate of hepatic gluconeogenesis in the intact mouse liver. *Cell Metabolism*.

[42] Gómez-Valadés A. G., Méndez-Lucas A., Vidal-Alabro A. (2008). *Pck1* gene silencing in the liver improves glycemia control, insulin sensitivity, and dyslipidemia in *db/db* mice. *Diabetes*.

[43] Ishii E. L., Bracht A. (1987). Glucose release by the liver under conditions of reduced activity of glucose 6-phosphatase. *Brazilian Journal of Medical and Biological Research*.

[44] Eler G. J., Peralta R. M., Bracht A. (2009). The action of n-propyl gallate on gluconeogenesis and oxygen uptake in the rat liver. *Chemico-Biological Interactions*.

[45] Youn Y. C., Kwon O. S., Han E. S., Song J. H., Shin Y. K., Lee C. S. (2002). Protective effect of boldine on dopamine-induced membrane permeability transition in brain mitochondria and viability loss in PC12 cells. *Biochemical Pharmacology*.

[46] de Souza Silva F. M., Rocha Alves da Silva M. H., Bracht A., Eller G. J., Constantin R. P., Yamamoto N. S. (2010). Effects of metformin on glucose metabolism of perfused rat livers. *Molecular and Cellular Biochemistry*.

[47] Hunter R. W., Hughey C. C., Lantier L. (2018). Metformin reduces liver glucose production by inhibition of fructose-1-6-bisphosphatase. *Nature Medicine*.

[48] Cermanova J., Prasnicka A., Dolezelova E. (2016). Pharmacokinetics of boldine in control and Mrp2-deficient rats. *Physiological Research*.

[49] Reagan-Shaw S., Nihal M., Ahmad N. (2008). Dose translation from animal to human studies revisited. *The FASEB Journal*.

